# The brain dynamics of trust decisions and outcome evaluation in narcissists

**DOI:** 10.3389/fpsyg.2022.929674

**Published:** 2022-08-05

**Authors:** Fengbo Guo, Ziyang Yang, Tengfei Liu, Li Gu

**Affiliations:** Key Laboratory for Quality of Life and Psychological Assessment and Intervention, School of Humanities and Management, Research Center for Quality of Life and Applied Psychology, Guangdong Medical University, Dongguan, China

**Keywords:** narcissism, trust, event-related potentials (ERP), N2, FRN

## Abstract

Individuals with narcissism are, by definition, self-centered, focus on self-benefit, and demonstrate less prosocial behaviors. Trusting strangers is risky, as it can result in exploitation and non-reciprocation. Thus, the trust may be antagonistic to narcissism. However, how narcissists make the choice to trust remains to be elucidated. The current study examined 44 participants (22 rated high in narcissism) playing as trustors in one-shot trust games, and their electroencephalograms were recorded. Individuals high in narcissism exhibited less trust toward strangers, especially following gaining feedback for their trust. In addition, narcissists exhibited a larger N2 following distrust and a stronger negatively-valanced difference in feedback-related negativity (dFRN) after trustee feedback. Our findings provide insights into how individuals with narcissism trust strangers. The results also shed light on the temporal course of brain activity involved in trust decision-making and outcome evaluation in individuals with narcissism.

## Introduction

Narcissism refers to a personality trait that is characterized by inflated self-view, egotistical, self-absorbed, dominant, and exploitive (Raskin and Hall, 1979; Jonason et al., 2012). Furthermore, instances of narcissism have increased in younger generations over the past decades in both the Western world and the Eastern world (Twenge and Foster, 2010; Cai et al., 2012). Narcissism comprises two forms: grandiose and vulnerable (Pincus et al., 2009; Miller et al., 2012). The former is characterized by exaggerated self-worth, entitlement exploitativeness, and feelings of superiority, while the latter is characterized by insecurity, anxiety, defensiveness, low self-esteem, and negative emotionality (Miller and Campbell, 2008). These two forms are sharing the common antagonistic core of entitlement and egotism (Crowe et al., 2019), and capture the pathological expression of narcissism (Krizan and Herlache, 2018; Crowe et al., 2019). Thus, we considered individuals who scored high on narcissism scales as “narcissists” in the current study.

Narcissists might be conditionally prosocial (i.e., help others when it benefits them, Eberly-Lewis and Coetzee, 2015), because acting prosocially strategically can help individuals secure status (Flynn et al., 2006). Narcissists typically focus on what benefits them, holding little regard for whether their actions benefit or harm others (Campbell and Foster, 2007). Moreover, narcissists often behave selfishly and immorally (Campbell et al., 2004; Brunell et al., 2014), manipulate others (Campbell and Foster, 2007; Foster and Trimm, 2008), emphasize short-term success over long-term cooperation (Malesza and Kaczmarek, 2018), and tend to ignore the future impact of their decisions, choosing smaller and immediate rewards instead of long-term gains (Jonason et al., 2010; Crysel et al., 2013). Therefore, it is difficult for narcissists to establish and maintain long-term interpersonal relationships (Back et al., 2010).

Trust is an individual’s willingness to be vulnerable to the actions of others based on the positive expectation of their behavior (Mayer et al., 1995) and may be antagonistic to narcissism (Miller et al., 2017). Narcissism is related to distrust since people high in narcissism are reluctant to make themselves vulnerable to others (Kong, 2015; Krizan and Johar, 2015; Poggi et al., 2019). Specifically, antagonistic narcissism (a form of narcissism that blends grandiose and vulnerable narcissism) is negatively related to general trust (Kwiatkowska et al., 2019; Szymczak et al., 2020). However, previous studies mainly focused on the trust belief. Trust belief is a cognitive perception about the attributes or characteristics of the trustee, which can shape one’s trust behavior (Fehr, 2009). Trust is the lubricant of social development (Fukuyama, 1995) and is regulated by an individual’s personality traits (Thielmann and Hilbig, 2015). Unfortunately, younger generations have become more narcissistic partly owing to social-cultural changes (e.g., social media use; McCain and Campbell, 2018), and individuals with narcissism demonstrate less prosocial behaviors (Brunell et al., 2014) and are less concerned with long-term relationships (Morf and Rhodewalt, 2001; Fossati et al., 2010). Thus, we hypothesized that people high in narcissism would be less trusting of strangers (*Hypothesis 1*), reflecting the focus of narcissists on self-benefit and selfish behavior.

In the current study, we investigated the behavior and brain activity of narcissists translated into trust or distrust of an unfamiliar person. Studies have shown that high narcissism exhibit distrust of others (Krizan and Johar, 2015; Poggi et al., 2019), but the mechanism behind this relationship remains unclear, especially with regard to the neural processes of narcissists when put into a position to trust strangers. We examined this issue by investigating the electrical brain activity of individuals who scored high or low in narcissism, while they played as trustors in a one-shot trust game (Berg et al., 1995) with unfamiliar trustees. We recorded their electroencephalograms (EEGs) with a high temporal resolution to capture brain activity related to trust/distrust decision-making and outcome evaluation.

Studies combining EEG and trust games have revealed that trustors exhibit a greater N2 following distrust than trust, which peaks at 250–350 ms after choice onset (Wang et al., 2016, 2017). The N2 is distributed in the frontocentral cortex and may be generated in the anterior cingulate cortex (ACC; Yeung et al., 2004), which has been linked with cognitive control processes involving conflict monitoring or response inhibition (Huster et al., 2013); a more negative N2 possibly reflects increased cognitive conflict detection (Folstein and Van Petten, 2008). In trust games, distrust elicited a more negative-going N2 compared to trust, which could be due to distrust decisions inducing greater cognitive conflict (Wang et al., 2016, 2017). Narcissists are driven by a dominant status motive (Grapsas et al., 2020), while distrust violates the social norm of cooperation and may be harmful to establishing and maintaining long-term friendships, which may be opposite to the dominant status and require greater cognitive control for narcissists. Thus, we expected that the N2 following distrust would be more prominent for individuals who scored high in narcissism (*Hypothesis 2*). Moreover, P3 is an important index of prosocial decisions that peaks at 300–400 ms after stimulus, as more motivational significant stimuli elicit a greater P3 (Nieuwenhuis et al., 2005). Thus, trusting decisions may elicit a greater P3 compared to distrust.

In addition, previous studies of outcome evaluation in trust games have suggested that loss (i.e., being exploited by others) induces increased feedback-related negativity (FRN) compared to gain (i.e., being reciprocated by others) after trust decisions (Wang et al., 2016, 2017). FRN is frontocentral negativity at 200–350 ms after feedback onset and may be generated in the ACC (Gehring and Willoughby, 2002) and striatum (Foti et al., 2011). FRN is often driven by unexpected errors or losses (Holroyd and Coles, 2002; Sambrook and Goslin, 2015). Greater loss FRN may indicate surprise in response to violated expectations when the stranger exploited and non-reciprocated the trustor (Wang et al., 2016, 2017). Narcissists are sensitive to social feedback from others (Morf and Rhodewalt, 2001) and experience unexpected losses more negatively (Thielmann and Hilbig, 2015). Thus, we expected that the FRN deviation between loss and gain would be stronger for high narcissism compared to low narcissism, as indicated by a larger difference in FRN (dFRN; *Hypothesis 3*). In addition, P300 is a positivity peak at 300–600 ms after a stimulus that is linked to the motivational-affective significance of outcomes, and positive or favorable outcomes elicit a more pronounced P300 (Wu and Zhou, 2009; Leng and Zhou, 2010). Thus, the gain feedback may elicit a greater P300 compared to the loss.

## Materials and methods

### Participants

A total of 315 undergraduates (72.06% women, 19.40 ± 1.13 years) completed the brief version of the Pathological Narcissism Inventory (B-PNI, Schoenleber et al., 2015). Previous studies have used the total scores to index participants’ pathological narcissism (Fossati et al., 2016; Mao et al., 2016), and the current study aimed to compare low and high narcissism; we selected the bottom and top 20% based on the total scores as a sample, resulting in 44 individuals who were recruited and agreed to volunteer in the study. Participants were divided into a high narcissism (HN) group (*n* = 22, 10 women) and a low narcissism (LN) group (*n* = 22, 14 women). All participants were right-handed, had a normal or corrected-to-normal vision, and none had a history of any neurological disorder. Each participant gave written informed consent before participation and received 20 RMB (about $3.2) in monetary compensation after the task was completed. This study was approved by the Guangdong Medical University Ethics Board.

### Behavioral task

The behavioral task was a trust game modified by Berg et al. (1995). Participants played as trustors with an alleged trustee, and both players were given 10 game points as an initial endowment. The trustor was required to decide whether to transfer the 10 points to the trustee or not. If the trustor chose to keep the points, the round was terminated, and the trustor received only their own 10 points; if the trustor decided to transfer the points, they were tripled to 30 points and given to the trustee. Following the transfer of points, the trustee was prompted to decide how to allocate their 40 points (30 transferred + the original 10); the trustee had the choice to divide his/her points equally or keep all 40 points. Considering the trustor’s transfer may be exploited by the trustee, this task is considered a behavioral operationalization of trust.

In the trust game utilized in the current study, participants were informed that the trustee was randomly selected from a pool of 400 adult subjects. These 400 adults were recruited from the local community and were asked the question “If you were trusted by a stranger and were given 30 points, how would you make the choice between sending 20 points back or keeping all 40 points?” and recorded their choices. We told the participants selected as trustors that we randomly selected from the subject pool’s response to their investment. All participants gave an evaluation of the trustees by answering the question “How do you think about the opponents in the game?” during the rest time and reported that they were playing with human partners after the experiment. In reality, the trustees’ responses were pre-programmed, with the rate of receiving 20 points following trust set to 50% for each participant. Thus, participants received the same final total points, resulting in identical monetary compensation.

### Stimuli and procedure

**FIGURE 1:**
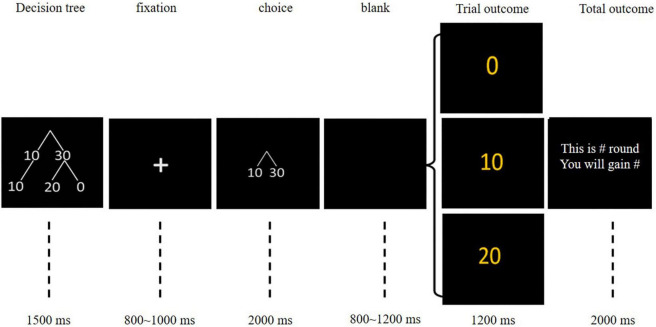
Sequence of each trial in the trust game.

Participants completed 150 rounds of the trust game, with a rest period after 75 rounds. As illustrated in Figure 1, in each round, participants first saw a simplified decision tree that showed possible decisions and outcomes for 1,500 ms. After the presentation of a fixation cross for 800–1,000 ms, the choice option was displayed for 2,000 ms. During this time, the participant was required to choose either to keep (cued by “10”) or to send (cued by “30”) their initial endowment (10 points) by pressing “1” or “3,” respectively. If the participant failed to respond, a “reacted too slowly” warning message was displayed, and that trial was repeated. The position of keeping and sending was counterbalanced between participants. Following an 800–1,200 ms black interval, the participant’s current trial score and the current total score were displayed for 1,200 and 2,000 ms, respectively. We provided 10 practice trials to ensure the participant completely understood the experimental procedure.

Upon the participant’s arrival, we first introduced the rules of the trust game. Then, the participant was fitted with an electrode cap and seated comfortably 1 m from a screen in an electromagnetically shielded room. The experiment commenced once the participant was ready.

### Electroencephalograms recording and analysis

We recorded brain electrical activity from 64 channels with an averaged bilateral mastoid reference and a forehead-ground using a modified 10–20 system electrode cap (Neuroscan Inc. Vctoria, Australia). A vertical electrooculogram was recorded with electrodes placed above and below the left eye; a horizontal electrooculogram was recorded with electrodes placed on the lateral canthus of both eyes; all the interelectrode impedance was kept below 5 kΩ. Biosignals were extracted with a 0.05–100 Hz bandpass filter and continuously sampled at 1,000 Hz/channel for offline analysis. Eyeblink artifacts were automatically removed *via* Scan software (Neurocan Inc.). All trials in which EEG voltages exceeded a threshold of ±75 μV during recording were regarded as artifacts and were excluded from further analysis. The average number of trials of decision (trust vs. distrust) and feedback (gain vs. loss) was: 68 ± 24, 52 ± 19, 34 ± 11, and 30 ± 11, respectively.

**FIGURE 2:**
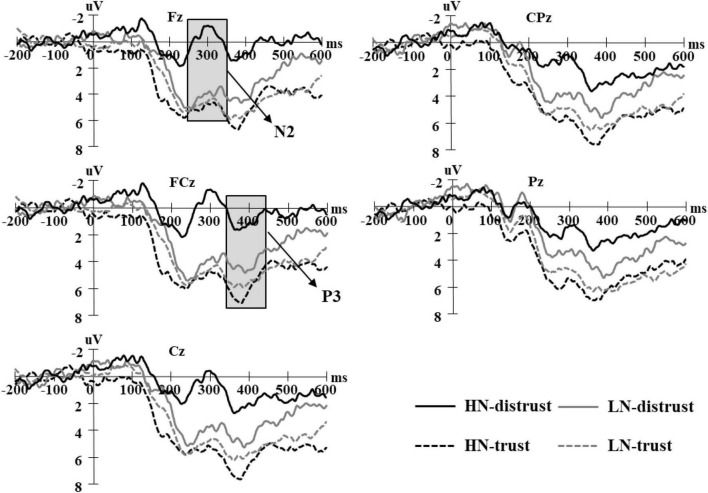
Grand average waveform for trusting and distrusting decision, separated by high narcissism (HN).

**FIGURE 3:**
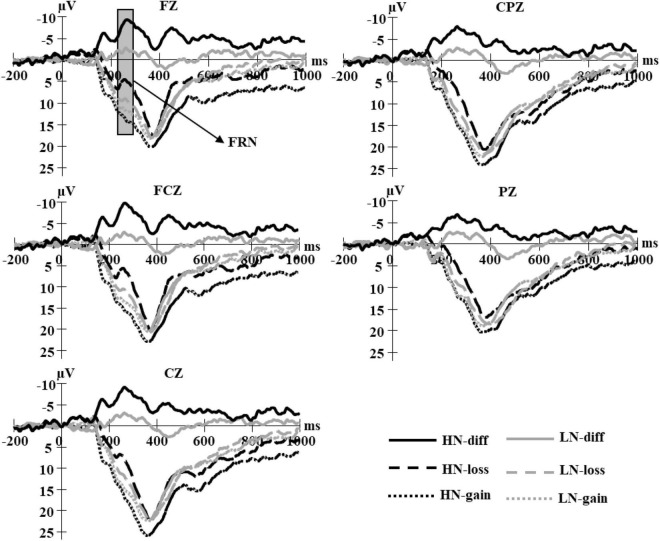
Grand average waveform for loss and gain feedback, separated by high narcissism (HN) and low narcissism (LN).

Decision epochs were extracted from 200 ms prior to 600 ms after choice onset, and the outcome epochs were extracted from 200 ms prior to 1,000 ms after feedback onset. We determined the time window for event-related potential (ERP) analysis through visual inspection of grand-averaged waveforms. Accordingly, we calculated the mean value within 230–340 ms for N2 and the mean value within 340–450 ms for P3 after choice onset in the decision stage (Figure 2). At the feedback stage, we calculated the mean value within 200–300 ms for FRN and the mean value within 300–600 ms for P3 after feedback onset; and to isolate FRN effects and minimize other component influences, we calculated the mean value within 200–300 ms for dFRN by subtracting gain trials from loss trials (Figure 3).

### Measurement

#### Brief version of pathological narcissism inventory (B-PNI)

The brief version of pathological narcissism inventory (B-PNI) is a 28-item self-report measure of pathological narcissism (Schoenleber et al., 2015). It consists of seven subscales and is further grouped into two factors; grandiosity is composed of grandiose fantasy, exploitativeness, and self-sacrificing self-enhancement; vulnerability is composed of contingent self-esteem, hiding of the self, devaluing, and entitlement rage. Items are rated on a 6-point Likert-type scale ranging from 1 (*not at all like me*) to 6 (*very much like me*). Higher scores reflect higher levels of pathological narcissism. The alpha coefficients for the B-PNI in our sample were 0.76–0.84.

#### Interpersonal reactivity index

The Chinese version of the interpersonal reactivity index (IRI; IRI-C) was used to assess participant empathy (Zhang et al., 2010), to exclude empathy as a possible confounding variable. It contains 22 items and is grouped into four subscales: perspective taking, empathic concern, personal distress, and fantasy. Items are rated on a 5-point Likert-type scale ranging from 1 (*does not describe me well*) to 5 (*describes me very well*). The alpha coefficients for the IRI-C in our sample ranged from 0.67 to 0.77.

#### General risk aversion scale

The General Risk Aversion Scale (GRAS; Mandrik and Bao, 2005) was used to assess participant risk aversion. This measure contains six-item respondents’ answers using a 7-point Likert scale ranging from 1 (*strongly disagree*) to 7 (*strongly agree*). Higher scores indicate greater risk aversion. The alpha coefficient for the GRAS in our sample was 0.72.

#### General trust scale

The General Trust Scale (GTS; Yamagishi and Yamagishi, 1994) is a six-item measure of generalized trust. This measure contains respondents’ answer using a 7-point Likert scale ranging from 1 (*strongly disagree*) to 7 (*strongly agree*). Higher scores indicate greater general trust. The alpha coefficient for the GTS in our sample was 0.82.

## Results

### Demographic and questionnaire data

Table 1 shows the demographic and questionnaire data for HN and LN groups. HN scored higher than LN on B-PNI and GRAS, *ps* < 0.001, *ds* > 0.68, and lower than LN on GTS, *p* < 0.05, *d* = 0.95. Meanwhile, no differences were noted between groups in age or IRI, *ps* > 0.05. Considering that general risk aversion and general trust had a group difference, to exclude these factors as possible confounding variables, we make these factors covariates in the behavioral and ERP analyses.

**TABLE 1:** Sample characteristics (*M* ± *SD*).

	LN	HN	*t*	*p*
Age	19.50 ± 1.06	19.64 ± 1.09	0.42	0.676
B-PNI	69.50 ± 5.65	108.73 ± 9.52	16.62	<0.001
IRI	73.27 ± 7.20	75.55 ± 6.91	1.07	0.292
GRAS	26.09 ± 5.73	29.77 ± 5.02	2.27	0.029
GTS	34.36 ± 5.13	29.45 ± 5.23	3.14	0.003

### Behavioral results

The average percentages of trust choices in HN (51.95 ± 15.77%) were lower than LN (63.23 ± 9.90%), *t*(42) = 2.84, *p* < 0.01, *d* = 0.86. Regression analysis revealed that narcissism significantly and negatively predicted trust choices (β = −0.44, *p* < 0.05) after controlling for general risk aversion and general trust. For the trust choices after different feedback (loss vs. gain), the group-by-feedback interaction was significant, *F*(1,40) = 4.96, *p* < 0.05, η_*p*_^2^ = 0.11. HN (38.82 ± 19.95%) made fewer trust choices than LN (51.00 ± 13.96%) after gain, *F*(1,40) = 4.96, *p* < 0.05, η_*p*_^2^ = 0.11. However, trust choices after loss were more common in HN (61.23 ± 19.95%) than in LN (49.00 ± 13.96%), *F*(1,40) = 4.97, *p* < 0.05, η_*p*_^2^ = 0.11. In addition, HN chose to be less trusting after gain than loss, *F*(1,40) = 8.67, *p* < 0.01, η_*p*_^2^ = 0.18, while there was no difference in LN (*p* > 0.05).

### Event-related potential results

#### Decision stage

##### N2

A 2 (group: HN vs. LN) × 2 (decision: trust vs. distrust) × 5 (electrode: Fz/FCz/Cz/CPz/Pz) mixed-model ANOVA was conducted on average N2 amplitudes (Figure 2). The interaction among group, decision, and electrode was significant, *F*(4,160) = 8.0, *p* < 0.01, η_*p*_^2^ = 0.17. The N2 was more negative for HN than for LN during distrust on Fz, FCz, and Cz (*ps* < 0.05); however, N2 for HN did not differ significantly from LN while exhibiting trust on any electrode (*ps* > 0.05). Meanwhile, the N2 for distrust was more negative than for trust in HN on any electrode (*ps* < 0.001), but there was no difference in LN (*ps* > 0.05).

##### P3

A 2 (group: HN vs. LN) × 2 (decision: trust vs. distrust) × 5 (electrode: Fz/FCz/Cz/CPz/Pz) mixed-model ANOVA was conducted on P3 average amplitudes (Figure 2). The interaction among group, decision, and electrode was significant, *F*(4,160) = 3.29, *p* < 0.05, η_*p*_^2^ = 0.08. The P3 during distrust was larger in LN than in HN on Fz, FCz, and Cz (*ps* < 0.05), whereas the P3 for trust demonstrated no significant difference between groups (*ps* > 0.05). Meanwhile, the P3 for trust was larger than distrust in HN on any electrode (*ps* < 0.01), but there was no difference in LN (*ps* > 0.05).

#### Feedback stage

##### Feedback-related negativity

A 2 (group: HN vs. LN) × 2 (feedback: loss vs. gain) × 5 (electrode: Fz/FCz/Cz/CPz/Pz) mixed-model ANOVA was conducted on FRN mean amplitudes (Figure 3). The group-by-feedback interaction was significant, *F*(1,40) = 22.68, *p* < 0.001, η_*p*_^2^ = 0.36. The FRN for loss (*M* = 5.86, *SE* = 1.18 μV) was smaller than that for gain (*M* = 12.89, *SE* = 1.21 μV) in HN, *F*(1,40) = 83.90, *p* < 0.001, η_*p*_^2^ = 0.68, whereas this difference (*M*_*loss*_ = 10.51, *SE* = 1.18 μV; *M*_*gain*_ = 12.01, *SE* = 1.21 μV) was marginally significant in LN, *F*(1,40) = 3.87, *p* = 0.056, η_*p*_^2^ = 0.09. That is, the FRN for loss was smaller in HN than in LN, *F*(1,40) = 6.76, *p* < 0.05, η_*p*_^2^ = 0.15, but the FRN for gain has no group difference (*ps* > 0.05).

##### Difference in feedback-related negativity

A 2 (group: HN vs. LN) × 5 (electrode: Fz/FCz/Cz/CPz/Pz) mixed-model ANOVA was conducted on the dFRN average amplitudes (Figure 3). The main effect of group was significant, and the dFRN for HN (*M* = -7.02, *SE* = 0.77 μV) was more negative than that for LN (*M* = -1.51, *SE* = 0.77 μV), *F*(1,40) = 22.68, *p* < 0.001, η_*p*_^2^ = 0.36. In addition, the group-by-electrode interaction was also significant, *F*(4,160) = 6.16, *p* < 0.01, η_*p*_^2^ = 0.13. The dFRN was largest at FCz and became smaller at the posterior sites for the HN group (*ps* < 0.05), but there was no difference on any electrode for the LN group (*ps* > 0.05).

##### P300

A 2 (group: HN vs. LN) × 2 (feedback: loss vs. gain) × 5 (electrode: Fz/FCz/Cz/CPz/Pz) mixed-model ANOVA was conducted on P3 mean amplitudes (Figure 3). The group-by-feedback interaction was significant, *F*(1,40) = 10.45, *p* < 0.01, η_*p*_^2^ = 0.21. The P3 amplitude for gain (*M* = 15.62, *SE* = 1.32 μV) was larger than that for loss (*M* = 11.71, *SE* = 1.50 μV) in HN, *F*(1,40) = 15.90, *p* < 0.001, η_*p*_^2^ = 0.28, but there was no difference in LN, *F*(1,40) = 0.38, *p* = 0.02.

## Discussion

In the existing literature, narcissism is characterized by a lack of trust in others (Kwiatkowska et al., 2019; Poggi et al., 2019; Szymczak et al., 2020). However, these studies mainly examined the trust belief and less regarded trust behavior. In addition, trust belief can shape one’s trust behavior (Fehr, 2009). To fill this gap, we investigated the behavioral performance and brain activity of narcissists in trust games to clarify the mechanism by which narcissists decide whether or not to trust strangers.

Trust is a risky social gamble; trusting strangers with an unknown reputation may result in a loss of self-interest. In the rational economic view, trustors transfer their endowment in the hope of obtaining monetary incentives from trustees’ reciprocity. However, the trustees may keep all of the endowments to maximize their own winnings. Therefore, trusting strangers is risky. In the current study, HN individuals reported higher risk aversion and lower general trust, suggesting that narcissists would transfer less endowment to avoid being exploited by trustees. Previous studies have revealed that narcissists are less willing to provide voluntary services (Brunell et al., 2014) or spend time helping others selflessly (Lannin et al., 2014). In addition, choosing trust in the trust game means giving over control of the outcome to the trustee, which might conflict with the narcissist’s exaggerated need for power (Morf and Rhodewalt, 2001) and dominance motivation (Johnson et al., 2012). Therefore, HN individuals’ tendency to exhibit less trust toward strangers may be due to their self-centered focus, decreased prosociality, and dominance. Furthermore, narcissists are typically unconcerned with long-term relationships (Fossati et al., 2010), exhibit hostility and arrogance (Grafeman et al., 2015), and often have negative expectations of others’ behavior (Lewicki et al., 1998). Thus, when strangers reciprocate, the narcissist may doubt whether they will reciprocate again, leading them to choose distrust to prevent potential exploitation.

High narcissism (HN) individuals exhibited a more negative N2 following distrust compared to trust; however, this N2 effect was not observed in LN individuals. In addition, the N2 elicited by distrust was more negative in HN than in LN. N2 is related to cognitive control (i.e., conflict monitoring; Huster et al., 2013) and is generated in the ACC (Yeung et al., 2004), which may be an important region for evaluating cost-benefit information (Holroyd and Yeung, 2012; Kolling et al., 2016). Previous studies have revealed that distrust elicits a more negative N2 and induces greater cognitive conflict compared to trust (Wang et al., 2016, 2017). Narcissists are driven by a dominant status motive (Grapsas et al., 2020) and typically focus on self-benefits (Campbell and Foster, 2007). Although distrust decisions exhibit dominance and have a certain benefit, they violate the cooperation social norms. Thus, HN individuals may require greater cognitive control to distrust compared to LN individuals, and these individuals may also require greater cognitive control to distrust when compared to trust. Furthermore, this N2 effect also sheds light on the self-regulation models of narcissism (Campbell and Foster, 2007), and effective self-regulation requires the capabilities of monitoring conflicts, the HN individuals exert greater cognitive control to obtain self-benefit. However, LN individuals may exert a certain extent of self-control in both trust and distrust; that is, trust is risky, while distrust violates cooperative norms, and both processes may involve the same or similar cognitive control.

Moreover, we found that HN individuals and LN individuals can also be distinguished based on the P3 associated with the distrust/trust decision. Particularly, the HN individuals exhibited a more positive P3 following trusting decisions compared to distrust decisions. Furthermore, the P3 elicited by distrust was smaller in both HN individuals and LN individuals. In the ERP literature, P3 is an important index of prosocial decision-making, which has been commonly linked with attention and motivation, as attention-capturing or motivational significant stimuli elicit a greater P3 (Nieuwenhuis et al., 2005). Our findings suggest that narcissists may attend more to trusting decisions (compared to distrust) that have greater social significance to them.

In the outcome evaluation, loss elicited a more negative FRN than gain, and this effect was more prominent in HN individuals compared to LN individuals, especially in the loss feedback. Furthermore, the HN individuals exhibited a more negative dFRN (losses minus gains) than LN individuals. Previous studies have linked FRN with reward prediction error (Sambrook and Goslin, 2015). From the economically rational view, a negative-trending dFRN associated with trust suggests that trust is based on the trustor’s motive to gain monetary reward; suffering loss from being trusting may violate one’s reward expectation (Chen et al., 2012). Narcissism is self-exaggerated and sensitive to social feedback (Morf and Rhodewalt, 2001). Thus, when HN individuals trust strangers, their expectation of trustees’ reciprocity may be more intense. If their expectation is not satisfied (i.e., they are exploited by strangers), HN individuals may experience greater expectation deviation and exhibit a more negative-trending dFRN. Error monitoring and action updating is the core of the self-regulation process, a more negative dFRN may reflect the HN individuals updating their thoughts, and actions to resolve conflicts. However, LN individuals may hold betrayal anticipation and a weaker expectation of trustees’ reciprocity; thus, they have blunted neural responses to betrayal outcomes.

High narcissism (HN) individuals also exhibited a larger P300 in gain when compared to loss feedback. P300 is linked to the motivational-affective significance of outcomes (Ferrari et al., 2011; Righi et al., 2012; Pfabigan et al., 2014), with more positive outcomes eliciting a more pronounced P300 (Wu and Zhou, 2009; Leng and Zhou, 2010). Furthermore, the P300 can also differentiate favorable outcomes from unfavorable outcomes during reward evaluation (Wu et al., 2012; Gu et al., 2018). The gained feedback in the trust game indicated to the trustor that the transfer of the endowment to the trustee was reciprocated. A previous study revealed that mutual benefit induced a larger P300 during interpersonal cooperation (Bai et al., 2014). Thus, trust and related reciprocity may have a higher motivational-affective significance for narcissists.

## Conclusion

The current study investigated the trust behavior and brain activity of narcissists. Our results showed that narcissists exhibited less trust in strangers in social interactions and that the decision to distrust requires more cognitive capacity, as there is ambivalence in response to the violation of social norms by the trustee and the possibility of a sullied social reputation for the narcissist. Our findings hint at narcissists exhibit some dysfunction in social interaction and suggest that we should identify the individuals with narcissistic tendencies and exert some intervention as early, to gain a smoothly social interaction.

## Limitations and future directions

In our study, the narcissists made trust decisions without any information about the trustees. Future studies should provide more information regarding the trustees; it is possible that people high and low in narcissism will be trusting strategically if the trustee is more familiar. In addition, we created subgroups of high and low narcissism based on overall scores on the Pathological Narcissism Inventory. Although the overall scores reflect the level of pathological narcissism, researchers believe narcissistic grandiosity and vulnerability are distinct constructs. Future studies should also distinguish between different forms of narcissism or treat narcissism as a continuous construct.

## Data availability statement

The original contributions presented in this study are included in the article. Further inquiries can be directed to the corresponding author.

## Ethics statement

The study involving human participants were reviewed and approved by Guangdong Medical University. All participants provided their written informed consent.

## Author contributions

FG collected and analyzed the data and drafted the manuscript. ZY and TL assisted with data interpretation and manuscript revision. LG conceived, designed, directed the study, and finally revised and approved the manuscript. All authors contributed to the article and approved the submitted version.
